# SECrackSeg: A High-Accuracy Crack Segmentation Network Based on Proposed UNet with SAM2 S-Adapter and Edge-Aware Attention

**DOI:** 10.3390/s25092642

**Published:** 2025-04-22

**Authors:** Xiyin Chen, Yonghua Shi, Junjie Pang

**Affiliations:** School of Mechanical and Automotive Engineering, South China University of Technology, Guangzhou 510640, China; 201910100254@mail.scut.edu.cn (X.C.); 202320100560@mail.scut.edu.cn (J.P.)

**Keywords:** crack segmentation, UNet, SAM2, edge-aware attention

## Abstract

Crack segmentation is essential for structural health monitoring and infrastructure maintenance, playing a crucial role in early damage detection and safety risk reduction. Traditional methods, including digital image processing techniques have limitations in complex environments. Deep learning-based methods have shown potential, but still face challenges, such as poor generalization with limited samples, insufficient extraction of fine-grained features, feature loss during upsampling, and inadequate capture of crack edge details. This study proposes SECrackSeg, a high-accuracy crack segmentation network that integrates an improved UNet architecture, Segment Anything Model 2 (SAM2), MI-Upsampling, and an Edge-Aware Attention mechanism. The key innovations include: (1) using a SAM2 S-Adapter with a frozen backbone to enhance generalization in low-data scenarios; (2) employing a Multi-Scale Dilated Convolution (MSDC) module to promote multi-scale feature fusion; (3) introducing MI-Upsampling to reduce feature loss during upsampling; and (4) implementing an Edge-Aware Attention mechanism to improve crack edge segmentation precision. Additionally, a custom loss function incorporating weighted binary cross-entropy and weighted IoU loss is utilized to emphasize challenging pixels. This function also applies Multi-Granularity Supervision by optimizing segmentation outputs at three different resolution levels, ensuring better feature consistency and improved model robustness across varying image scales. Experimental results show that SECrackSeg achieves higher precision, recall, F1-score, and mIoU scores on the CFD, Crack500, and DeepCrack datasets compared to state-of-the-art models, demonstrating its excellent performance in fine-grained feature recognition, edge segmentation, and robustness.

## 1. Introduction

Cracks in concrete structures are one of the common manifestations of building damage and play an important role in indicating structural safety, especially in the long-term health monitoring of bridges, tunnels, and high-rise buildings. According to relevant research, the comprehensive estimated cost of traffic accidents caused by road conditions in the United States in 2006 was as high as 217.5 billion US dollars [1], and human inspection errors are considered one of the main causes of accident hazards. Current crack detection in engineering structures mainly relies on visual inspection and manual measurement, which are not only inefficient [2] but also prone to subjective bias and safety risks. Although digital image processing techniques (such as Sobel edge detection [3] and Canny operator [4]) have partially achieved automated detection, their high false detection rates in complex environments (such as shadows, stains, and uneven lighting) [5,6] make it difficult to meet the high-precision and robustness requirements of engineering practice. Therefore, developing precise and reliable automated crack detection technologies is of great significance for improving detection efficiency and accuracy and reducing safety risks.

With the development of deep learning, the research domain of crack detection has gradually transitioned from conventional image processing techniques to CNN-oriented methods [7]. These approaches acquire knowledge from numerous samples to autonomously extract crack features, accomplishing accurate identification and extraction of cracks. Popular deep learning-driven image segmentation algorithms encompass Fully Convolutional Networks (FCNs) [8], U-Net [9], U-Net++ [10], DeepLabv3 [11], and DeepLabv3+ [12]. Among these, FCNs realize end-to-end pixel-level segmentation through the elimination of fully connected layers in traditional CNNs. U-Net gains recognition for its lightweight encoder-decoder framework and skip connections. DeepLab remarkably enhances segmentation precision via dilated convolutions and multi-scale feature integration, being extensively applied in the detection of slender objects, particularly within the crack detection field.

Deep learning has significantly advanced crack detection but still faces notable challenges. Traditional CNNs frequently overlook micro-cracks and lose high-frequency details during upsampling, resulting in discontinuous crack edges. Limited samples also pose a risk of overfitting. Recent GNN advancements, such as Cai et al.’s MDGMIN [13] and Zhao et al.’s MPGCTN [14], demonstrate promise in managing imbalanced data and enhancing diagnostic precision. However, deep learning continues to struggle with the diverse shapes of real-world cracks, underscoring the need for further innovation in crack detection techniques.

In recent years, the Transformer architecture has obtained remarkable success in natural language processing and has progressively been implemented in computer vision tasks. Vision Transformer (ViT) [15] and Swin Transformer (hierarchical vision transformer adopting shifted windows) [16] have efficiently captured long-range dependencies in images via self-attention mechanisms, offering novel concepts for image segmentation tasks. For instance, models like OCRNet [17] and Attention UNet [18] integrate the Transformer with U-Net, fully leveraging the Transformer’s global modeling strengths and U-Net’s local feature extraction advantages, thus notably enhancing segmentation accuracy. CT-crackseg [19] has notably enhanced the accuracy and robustness of crack detection by incorporating 3D CT scan data, multi-scale feature extraction, convolutional neural networks, and the Transformer architecture, addressing issues like edge blurring and small crack missed detection. Nevertheless, the intricate and diverse shapes of cracks on real structural surfaces still present challenges to deep learning methods in crack detection:

- Insufficient samples can weaken the generalization ability of deep learning models, making them prone to overfitting the training data and thus unable to accurately detect cracks in different scenarios in practical applications.
- Fine-grained feature loss: Traditional convolutional neural networks (CNNs) tend to ignore micro-crack textures during deep feature extraction, leading to missed detections of small targets.
- Edge blurring issue: Standard bilinear upsampling or deconvolution operations lead to the loss of high-frequency details during resolution recovery, resulting in discontinuous crack boundary segmentation.
- Insufficient edge perception: Small cracks are difficult to highlight, and edge details are easily overlooked.

The emergence of visual foundation models has brought great potential to the field of image segmentation. Among them, the Segment Anything Model (SAM1) [20] and its successor Segment Anything 2 (SAM2) [21] are particularly prominent. SAM2 has been optimized based on SAM1 by using a larger training dataset and improved architectural design. However, even with these improvements, SAM2 still generates category-agnostic segmentation results without manual prompts, highlighting the ongoing challenge of effectively applying it to downstream tasks that typically require task-specific or category-specific segmentation. The introduction of the hierarchical backbone in SAM2 provides new possibilities for designing more effective U-shaped networks, which offers potential solutions to the aforementioned problems.

As a new paradigm in artificial intelligence, foundation models are typically based on large-scale pre-trained Transformer architectures and have billions of parameters [22,23]. In the field of computer vision, SAM2, as a semantic segmentation model, has been trained on large-scale datasets and can adapt to various tasks in a zero-shot manner. However, direct application to crack segmentation is not feasible because SAM2 tends to generate masks for all distinguishable instances and performs poorly in recognizing fine cracks. Training such a large model directly requires enormous computational resources and data, which is unrealistic for many specific tasks. Therefore, fine-tuning SAM2 to learn the specific semantics of cracks is particularly important.

To address these issues, this paper proposes the SECrackSeg model, which adopts the following innovative measures:

- SAM2 S-Adapter: By freezing the 214M-parameter backbone of the Segment Anything Model 2 (SAM2) and fine-tuning only lightweight S-Adapter modules, this parameter-efficient design mitigates overfitting insufficient samples while retaining generalization capabilities from SAM2’s large-scale pretraining.
- MSDC (Multi-Scale Dilated Convolution) module: Promotes multi-scale feature fusion and improves the segmentation accuracy of cracks of different sizes.
- MI-Upsampling: Integrates slice upsampling, bilinear interpolation, and deconvolution operations to reduce information loss during resolution recovery.
- Edge-Aware Attention Mechanism: Enhances the segmentation accuracy of crack edges.

The structure of this paper is as follows: Section 2 introduces the architecture of the SECrackSeg model and elaborates on the design philosophy of the SAM2 S-Adapter, MSDC module, MI-Upsampling, Edge-Aware Attention mechanism, and custom Loss. Section 3 presents the experimental results, including performance evaluations on the three public datasets, as well as ablation studies. Section 4 concludes the paper by summarizing the advantages of the SECrackSeg model and future research directions.

## 2. Methods

**Figure 1:**
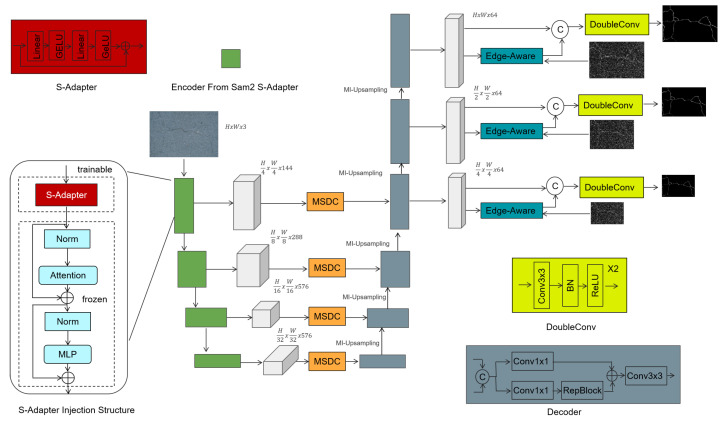
Architecture of the SECrackSeg model, including SAM2 S-Adapter, MSDC module, MI-Upsampling, and Edge-Aware Attention mechanism.

The SECrackSeg model is based on an improved U-Net architecture and deeply integrates the general segmentation capabilities of the Segment Anything Model 2 (SAM2) with edge-aware attention technology. The overall architecture is shown in Figure 1 and consists of five main parts: the encoder based on the SAM2 S-Adapter, the multi-scale dilated convolution module with skip connections, the decoder, hybrid upsampling, and the edge enhancement module. Here is a detailed description of each module:

### 2.1. Encoder Based on the SAM2 S-Adapter

In the field of computer vision, especially in crack segmentation tasks where data scarcity is often a significant challenge, efficient and accurate feature extraction is of great significance. The encoder of the SECrackSeg model is designed based on the SAM2 S-Adapter, leveraging the advantages of lightweight adapters to effectively extract multi-scale features related to cracks from input images.

#### 2.1.1. Backbone Network of the Encoder

The main backbone network of the SECrackSeg model’s encoder adopts the Hiera vision encoder from SAM2 [24]. In small-sample scenarios, the multi-scale feature extraction ability of the Hiera encoder becomes even more crucial. It has powerful capabilities in image feature extraction and can output four levels of cascaded multi-scale feature maps. Specifically, the output feature maps can be expressed in the following mathematical form:

(1) $$X_{i}\in R^{C_{i}\times \frac{H}{2^{i+1}}\times \frac{W}{2^{i+1}}},C_{i}\in \left\{144,288,576,1152\right\}$$

Here, *H* and *W* represent the height and width of the input image, respectively. *i* represents the level of the feature map, ranging from 0 to 3. As the value of *i* increases, the spatial resolution of the feature map gradually decreases, that is, the height and width become $\frac{1}{2^{i+1}}$ of the original. And $C_{i}$ represents the number of channels of the feature map at each scale, which are 144, 288, 576, and 1152, respectively. This multi-scale feature representation can capture image information at different levels, providing a rich feature basis for subsequent crack segmentation tasks.

The Hiera encoder has a unique feature extraction mechanism, playing different roles at different levels. In the shallow layers of the network, the Hiera encoder mainly focuses on the edge details of cracks, such as the texture and direction of cracks. The shallow-layer feature maps have a high spatial resolution, which can retain the local information in the image, thus accurately capturing the subtle changes in the edges of cracks. For example, for some fine cracks, the shallow-layer features can precisely depict the outline and orientation of their edges.

In the deep layers of the network, the Hiera encoder focuses on extracting semantic context information, such as the branch structure of cracks. The deep-layer feature maps have a lower spatial resolution but more channels, which can integrate broader image information, thereby understanding the semantic relationships of cracks in the entire image. For instance, when there are branches in a crack, the deep-layer features can identify the connection relationships and the overall structural layout among these branches.

#### 2.1.2. Model Fine-Tuning Strategy

In practical applications, performing full-scale fine-tuning on the entire Hiera model, which contains as many as 214 M parameters, is not feasible due to memory limitations and computational resource constraints. To address this issue, researchers usually adopt a parameter-efficient fine-tuning strategy [25]. Specifically, most of the model’s parameters are frozen, and only a few key parts are adjusted. In the SECrackSeg model, we choose to insert adapter modules at key positions and fine-tune the parameters of these adapter modules to achieve efficient fine-tuning of the model.

The S-Adapter module is one of the core components of the SECrackSeg model’s encoder and is inserted before each Hiera multi-scale block. The module consists of the following parts:

- **Linear Down-projection Layer**
  This layer compresses input features to a lower-dimensional space using a weight matrix $Linear_{\mathrm{down}}\left(\dim_{\mathrm{in}},\dim_{\mathrm{mid}}\right)$, where $\dim_{\mathrm{mid}}<\dim_{\mathrm{in}}$. It reduces feature complexity, lowers computational cost, and helps the model focus on key information.
- **GeLU Activation Layer**
  The GeLU activation function introduces non-linearity, enhancing the model’s ability to fit complex features in crack images, such as irregular shapes and texture changes.
- **Linear Up-projection Layer**
  This layer restores the dimension of the compressed and transformed features to the original dimension using a weight matrix $Linear_{\mathrm{up}}\left(\dim_{\mathrm{mid}},\dim_{\mathrm{out}}\right)$, typically with $\dim_{\mathrm{out}}=\dim_{\mathrm{in}}$, ensuring compatibility with subsequent network layers.
- **Residual Connection**
  The residual connection preserves original feature information and prevents gradient vanishing by directly adding the input feature $X_{in}$ to the transformed feature, ensuring gradient stability during backpropagation.

The calculation process of the S-Adapter module can be expressed by the following formula:

(2) $$X_{\mathrm{out}}=X_{\mathrm{in}}+\mathrm{GeLU}\left(Linear_{\mathrm{up}}\left(\dim_{\mathrm{mid}},\dim_{\mathrm{out}}\right)\cdot \mathrm{GeLU}\left(Linear_{\mathrm{down}}\left(\dim_{\mathrm{in}},\dim_{\mathrm{mid}}\right)\cdot X_{\mathrm{in}}\right)\right)$$

where $X_{in}$ is the input feature and $X_{out}$ is the output feature. $Linear_{down}\left(dim_{\mathrm{in}},dim_{\mathrm{mid}}\right)$ is the weight matrix of the linear down-projection layer, which is used to reduce the dimension of the input feature from $dim_{in}$ to $dim_{mid}$. $Linear_{up}\left(dim_{\mathrm{mid}},and\;dim_{\mathrm{out}}\right)$ is the weight matrix of the linear up-projection layer, which is used to restore the dimension of the feature from $dim_{mid}$ to $dim_{out}$. First, the input feature $X_{in}$ undergoes non-linear transformation through the linear down-projection layer and the first GeLU activation layer, then through the linear up-projection layer and the second GeLU activation layer, and finally, it is added to the original input feature $X_{in}$ through the residual connection to obtain the final output feature $X_{out}$. This design enables the S-Adapter module to effectively adjust and optimize the features while retaining the original feature information, thus improving the performance of the model in crack segmentation tasks.

### 2.2. Multi-Scale Dilated Convolution Module

**Figure 2:**
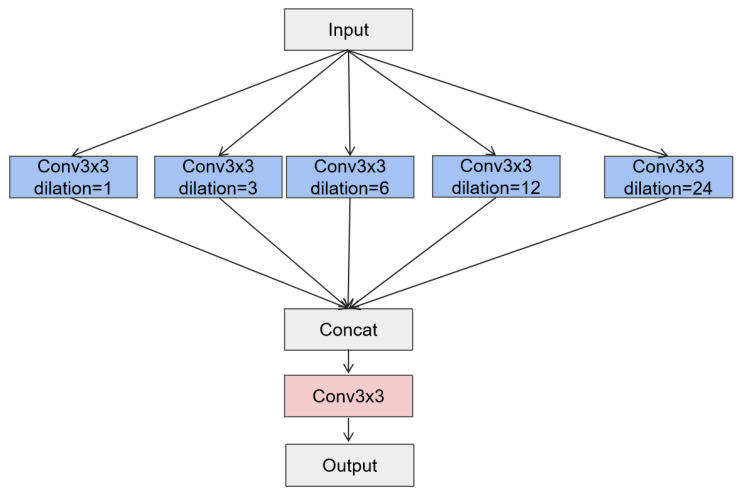
Multi-Scale Dilated Convolution (MSDC) module, including the parallel convolutional layers with varying dilation rates (1, 3, 6, 12, and 24).

The Multi-Scale Dilated Convolution (MSDC) module, designed in reference to [26], is a module used in convolutional neural networks that aims to capture multi-scale feature information through convolutional layers with different dilation rates. As shown in Figure 2, the module consists of multiple parallel convolutional layers, each with a kernel size of 3 × 3 and dilation rates of 1, 3, 6, 12, and 24. These layers enable the module to expand its receptive field without increasing the number of parameters, effectively capturing a wide range of contextual information. The outputs of all convolutional layers are concatenated into a feature map that fuses multi-scale information. Then, this fused feature map is passed through a 3 × 3 convolutional layer to further integrate features and reduce dimensions, resulting in the module’s output. The MSDC module enhances the network’s ability to segment objects of various sizes in images.

### 2.3. Decoder

SAM2’s initial mask decoder adopts a bidirectional transformer method to promote the interaction between prompt embeddings and encoder features. Differently, our decoder draws inspiration from the highly adaptable U-shaped structure—this structure has demonstrated effectiveness in numerous tasks [27,28] and adheres to the classic U-Net design. It comprises three decoder blocks, each including two “convolution–batch normalization–ReLU” combinations. Here, “convolution” signifies a 3 × 3 convolutional layer, while “batch normalization” stands for batch normalization operations.

### 2.4. Multi-Integration Upsampling

In the upsampling process within decoder blocks, resolution adjustment of feature maps serves as a critical step for achieving effective feature representation. Traditional upsampling approaches, such as bilinear interpolation or transposed convolution (deconvolution), are commonly utilized to elevate the resolution of feature maps. However, these methods may introduce blurring phenomena, affecting the model’s clarity and precision in detailing.

To overcome these limitations, inspired by the work in [29], we put forward a novel composite upsampling algorithm. This algorithm is engineered to maximize the retention of detail information while adjusting the resolution of feature maps, thereby enhancing the model’s accuracy.

**Figure 3:**
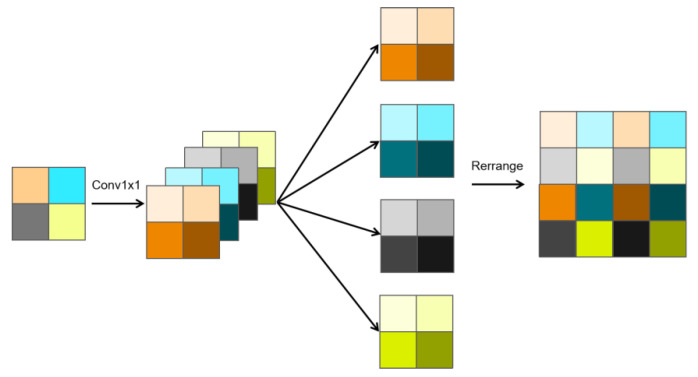
Slice Upsampling, including the steps of 1 × 1 convolution, channel separation, and rearrangement.

As shown in the Figure 3, the slice upsampling is designed with the following procedures:

1. **1 × 1 Convolution**: Generate a feature map with four times the number of channels through 1 × 1 convolution.
2. **Channel Separation**: Divide the channels into four segments, each possessing the same number of channels as the original feature.
3. **Rearrangement**: Map the corresponding points of the four segments to adjacent features, generating a feature map with twice the original width and height yet the same number of channels.

Furthermore, let the original input feature be *X*. The feature acquired through slice upsampling is $Y_{\mathrm{slice}}$; the feature obtained via bilinear interpolation is $Y_{\mathrm{bilinear}}$; and the feature derived from 3 × 3 deconvolution with a stride of 2 is $Y_{\mathrm{deconv}3\times 3}$. All three features ($Y_{\mathrm{slice}}$, $Y_{\mathrm{bilinear}}$, $Y_{\mathrm{deconv}3\times 3}$) are computed from the input feature *X*, ensuring that the final output feature $Y_{h}$ indirectly depends on *X*.

Next, concatenate $Y_{\mathrm{slice}}$, $Y_{\mathrm{bilinear}}$, and $Y_{\mathrm{deconv}3\times 3}$ along the channel dimension, and then reduce the number of channels back to the original number of channels of *X* through a 1 × 1 convolution, resulting in the final output feature $Y_{h}$.

The formula is as follows:

(3) $$Y_{h}=\mathrm{Conv}1\times 1\left(\mathrm{Concat}\left(Y_{\mathrm{slice}},Y_{\mathrm{bilinear}},Y_{\mathrm{deconv}3\times 3}\right)\right)$$

The final output feature $Y_{h}$ is used as the input to the next decoder block for further processing or passed to the Edge-Aware Attention Module (Section 2.5) to enhance edge details, depending on the stage in the decoder pipeline.

We name this series of composite upsampling operations MI-Upsampling. This method enhances the detail information of features through channel operations, rearrangement mechanisms, and multi-feature fusion, ensuring the model’s accuracy and reliability.

### 2.5. Edge-Aware Attention

**Figure 4:**
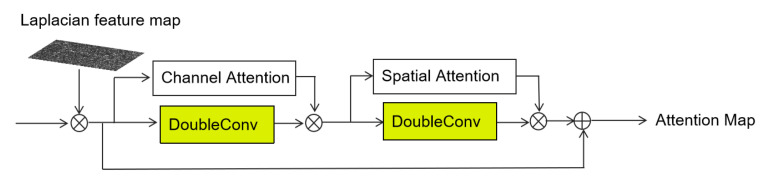
Edge-Aware Attention Module, composed of Laplacian-based edge extraction, two-stage attention-convolution fusion, and final residual enhancement.

As shown in Figure 4, the Edge-Aware Attention Module is designed to enhance crack boundaries and fine details by incorporating edge priors and a two-stage attention-convolution fusion strategy. The module consists of the following steps:

First, the Laplacian operator Δ [30] is applied to the input grayscale image *I* to extract edge information and produce a Laplacian feature map:

(4) $$L_{\mathrm{edge}}=\mathrm{ReLU}\left(|\Delta *I|\right)$$

where ∗ denotes the convolution operator, and |·| denotes the absolute value operation. The Laplacian kernel is defined as follows:

(5) $$\Delta =\left[\begin{array}{ccc} 0 & -1 & 0\\ -1 & 4 & -1\\ 0 & -1 & 0 \end{array}\right]$$

The resulting edge map is element-wise multiplied with the input feature map *F* to generate the fused feature map $F_{\mathrm{in}}$:

(6) $$F_{\mathrm{in}}=L_{\mathrm{edge}}⊙F$$

where ⊙ denotes element-wise multiplication.

Next, the fused feature $F_{\mathrm{in}}$ is processed by two parallel branches:

**(1) Channel Attention branch:** This branch calculates the channel attention weights α using global average pooling (GAP) and global max pooling (GMP), followed by a shared multilayer perceptron (MLP) [31]:

(7) $$\alpha =\sigma (\mathrm{MLP}\left(\mathrm{GAP}\left(F_{\mathrm{in}}\right)\right)+\mathrm{MLP}\left(\mathrm{GMP}\left(F_{\mathrm{in}}\right)\right))$$

where σ(·) is the Sigmoid activation function.

**(2) DoubleConv branch:** This branch applies two successive 3×3 convolutions, each followed by Batch Normalization (BN) and ReLU activation, to extract deep feature representations:

(8) $$F_{\mathrm{dc}1}=\mathrm{ReLU}\left(\mathrm{BN}\left(\mathrm{Conv}3\times 3\left(\mathrm{ReLU}\left(\mathrm{BN}\left(\mathrm{Conv}3\times 3\left(F_{\mathrm{in}}\right)\right)\right)\right)\right)\right)$$

The outputs of the above two branches are combined via element-wise multiplication:

(9) $$F_{1}=\alpha ⊙F_{\mathrm{dc}1}$$

Subsequently, the intermediate result $F_{1}$ is further enhanced by two new branches:

**(3) Spatial Attention branch:** This branch generates a spatial attention map β using channel-wise maximum and average pooling, followed by concatenation and convolution [31]:

(10) $$\beta =\sigma (W_{s}\cdot \mathrm{Concat}\left(\max\left(F_{1}\right),\mathrm{avg}\left(F_{1}\right)\right)+b_{s})$$

where max(·) and avg(·) denote the maximum and average pooling operations along the channel axis, respectively. $W_{s}$ and $b_{s}$ are learnable convolution kernel weights, and Concat(·) represents channel-wise concatenation. Here, $W_{s}$ and $b_{s}$ are learnable convolution kernel parameters. They are initialized randomly using a normal distribution and optimized during training through backpropagation. This optimization process aims to enhance spatial attention for crack edges, which is crucial for effectively capturing contour features and detail structures in the Edge-Aware Attention Module.

**(4) Another DoubleConv branch:** This branch again applies two 3×3 convolution layers with BN and ReLU to $F_{1}$:

(11) $$F_{\mathrm{dc}2}=\mathrm{DoubleConv}\left(F_{1}\right)$$

The two outputs are combined through element-wise multiplication:

(12) $$F_{2}=\beta ⊙F_{\mathrm{dc}2}$$

Finally, the module applies a residual connection by summing $F_{2}$ and the fused feature $F_{\mathrm{in}}$ to obtain the final attention map:

(13) $$AM=F_{2}+F_{\mathrm{in}}$$

Here, + denotes element-wise addition.

This two-stage fusion mechanism combines edge priors and hierarchical attention to effectively capture contour features and detail structures. The Edge-Aware Attention module is embedded in the final decoder and two upsampling stages, contributing to enhanced multi-scale edge representation and accurate crack localization.

Compared with conventional attention modules such as CBAM [31], our design introduces two major enhancements. First, we explicitly incorporate edge priors through a Laplacian operator, enabling the attention map to focus on boundary-sensitive regions. Second, we adopt a two-stage structure where both channel and spatial attention are reinforced by convolutional residual branches. This hierarchical fusion strategy provides stronger feature expressiveness for crack contours and discontinuities, which are often weak or fragmented in crack segmentation tasks.

### 2.6. Multi-Granularity Supervision and Loss Function

The standard binary cross-entropy loss $L_{\mathrm{BCE}}$ is defined as:

(14) $$L_{\mathrm{BCE}}=\sum_{i,j}\mathrm{BCE}\left(p_{i,j},g_{i,j}\right)$$

where $\mathrm{BCE}\left(p_{i,j},g_{i,j}\right)=-\left[g_{i,j}\cdot \log\left(p_{i,j}\right)+\left(1-g_{i,j}\right)\cdot \log\left(1-p_{i,j}\right)\right]$, $p_{i,j}$ is the predicted probability, and $g_{i,j}$ is the true label.

The standard IoU loss $L_{\mathrm{IoU}}$ is defined as:

(15) $$L_{\mathrm{IoU}}=1-\frac{\sum_{i,j}p_{i,j}\cdot g_{i,j}}{\sum_{i,j}\left(p_{i,j}+g_{i,j}-p_{i,j}\cdot g_{i,j}\right)}$$

Unlike the standard losses, the weighted binary cross-entropy loss $L_{\mathrm{BCE}w}$ and weighted IoU loss $L_{\mathrm{IoU}w}$ introduce a weighting factor to emphasize difficult pixels, particularly those near crack edges. Specifically, $L_{\mathrm{BCE}w}$ assigns higher weights to edge pixels, improving the model’s focus on fine details, while $L_{\mathrm{BCE}}$ treats all pixels equally, potentially missing subtle crack features. Similarly, $L_{\mathrm{IoU}w}$ enhances segmentation accuracy by prioritizing challenging regions, whereas $L_{\mathrm{IoU}}$ may lead to suboptimal performance in imbalanced datasets like crack segmentation.

The loss function in this paper, referencing the methods in references [32,33], is defined as follows:

(16) $$L=L_{\mathrm{IoU}w}+L_{\mathrm{BCE}w}$$

Here, $L_{\mathrm{IoU}w}$ and $L_{\mathrm{BCE}w}$ stand for the weighted IoU loss and the weighted binary cross-entropy (BCE) loss, applied for global and local (pixel-level) constraints, respectively. Different from the standard IoU loss commonly used in segmentation tasks, the weighted IoU loss highlights the significance of difficult pixels by increasing their weights. Moreover, compared with the standard BCE loss, $L_{\mathrm{BCE}w}$ focuses more on difficult pixels, whose effectiveness has been verified in the salient object detection field.

Specifically, the weighted binary cross-entropy loss $L_{\mathrm{BCE}w}$ is defined as follows:

(17) $$L_{\mathrm{BCE}w}=\sum_{i,j}w_{i,j}\cdot \mathrm{BCE}\left(p_{i,j},g_{i,j}\right)$$

In this formula: $p_{i,j}$ is the predicted probability value; $g_{i,j}$ is the true label value; $w_{i,j}=1+5\cdot \left|\mathrm{AvgPool}\left(g_{i,j}\right)-g_{i,j}\right|$ indicates the weight of the edge region, with weights closer to the edge being higher; $\mathrm{BCE}\left(p_{i,j},g_{i,j}\right)=-\left[g_{i,j}\cdot \log\left(p_{i,j}\right)+\left(1-g_{i,j}\right)\cdot \log\left(1-p_{i,j}\right)\right]$ is the binary cross-entropy loss.

The weighted IoU loss $L_{\mathrm{IoU}w}$ is defined as follows:

(18) $$L_{\mathrm{IoU}w}=1-\frac{\mathrm{Intersection}+1}{\mathrm{Union}-\mathrm{Intersection}+1}$$

where $\mathrm{Intersection}=\sum_{i,j}w_{i,j}\cdot p_{i,j}\cdot g_{i,j}$ is the weighted intersection of the predicted and true values; $\mathrm{Union}=\sum_{i,j}w_{i,j}\cdot \left(p_{i,j}+g_{i,j}\right)$ is the weighted union of the predicted and true values. In this paper, deep supervision is conducted on three different resolution outputs. The total loss of the proposed SECrackSeg is expressed as follows:

(19) $$L_{\mathrm{total}}=\sum_{i=1}^{3}L\left(G_{i},S_{i}\right)$$

where $G_{i}$ is the different resolution of the round truth segmentation mask, and $L(G_{i},S_{i})$ represents the loss of the *i*-th resolution output compared to the ground truth.

## 3. Results and Discussion

### 3.1. Datasets

For crack detection, several datasets have been created to assess the performance of different algorithms. This paper employs three datasets, detailed as follows:

**CFD Dataset [34]**

Collected in Beijing with an iPhone 5, the CFD dataset comprises 118 RGB road images (480 × 320 pixels), including 250 training, 50 validation, and 200 test images. It incorporates noise elements like oil stains, shadows, and water stains, focusing solely on road surface texture and cracks while excluding unrelated objects (e.g., garbage, cars). The diverse noise and environments pose challenges for crack detection algorithms, simulating real urban road conditions.

**Crack500 Dataset [35]**

Comprising 500 images (resolution near 2000 × 1500 pixels) taken via mobile phones on Temple University’s campus, the Crack500 dataset adapts to computational limits by dividing each image into 16 non-overlapping parts. Only parts with over 1000 crack pixels are retained. After meticulous pixel-level annotation, it now includes 3368 crack images.

**DeepCrack Dataset [36]**

With 537 crack images, DeepCrack is characterized by complex backgrounds and multi-scale cracks, covering three textures (bare, dirty, rough) and two scenarios (concrete, asphalt). Cracks vary in width (1–180 pixels), with small crack areas in each image—reflecting real conditions. Hand-annotated, they form binary representations.

These datasets offer a comprehensive evaluation of the SECrackSeg model across common real-world scenarios. Their diversity and complexity ensure thorough testing of the model’s performance.

### 3.2. Experimental Setup

Experiments were conducted on a Windows 10 system with an RTX 2070 Super GPU. We used Python 3.8.20, achieving GPU acceleration through CUDA 11.8 and CUDNN 8.9, implementing deep learning based on the PyTorch framework.

To comprehensively evaluate the semantic segmentation model, key metrics—Precision, Recall, F1-Score, and Mean Intersection over Union (mIoU)—were adopted. These metrics evaluate the model from multiple perspectives, helping to understand its advantages and disadvantages in different tasks. Their definitions and formulas are as follows:

**Precision**

Precision measures the proportion of truly positive samples among those predicted as positive by the model. The formula is:

(20) $$\mathrm{Precision}=\frac{TP}{TP+FP}$$

Here, TP represents True Positives (pixels predicted as positive and actually positive), and FP is False Positives (pixels predicted as positive but actually negative).

**Recall**

Recall evaluates the model’s ability to identify all actual positive samples, defined by:

(21) $$\mathrm{Recall}=\frac{TP}{TP+FN}$$

where FN denotes False Negatives (pixels predicted as negative but actually positive).

**F1-Score**

F1-Score, the harmonic mean of Precision and Recall, considers both prediction precision and coverage, suitable for imbalanced class scenarios. Its formula is:

(22) $$F1\text{-}\mathrm{Score}=\frac{2\cdot \mathrm{Precision}\cdot \mathrm{Recall}}{\mathrm{Precision}+\mathrm{Recall}}$$

**Mean Intersection over Union (mIoU)**

mIoU measures model performance across all classes by averaging the Intersection over Union (IoU) for each class. For the *i*-th class, IoU is:

(23) $${\mathrm{IoU}}_{i}=\frac{TP_{i}}{TP_{i}+FP_{i}+FN_{i}}$$

With $TP_{i}$, $FP_{i}$, $FN_{i}$ as True Positives, False Positives, and False Negatives for the *i*-th class. mIoU is:

(24) $$\mathrm{mIoU}=\frac{1}{C}\sum_{i=1}^{C}{\mathrm{IoU}}_{i}$$

where *C* is the number of classes.

These metrics reflect the model’s strengths and weaknesses from different angles. Using them together enables a comprehensive performance evaluation, providing a basis for model optimization and selection.

### 3.3. Evaluation of SECrackSeg

To demonstrate the effectiveness of our proposed model, we used several mainstream crack segmentation methods as our baselines.

Before the comparison, we provided some relevant explanations for these mainstream algorithms:

- **UNet [9]**: A widely used U-shaped convolutional neural network for image segmentation.
- **UNet++ [10]**: An improved version of UNet with a nested U-shaped architecture.
- **DeepLabv3+ [11]**: A state-of-the-art semantic segmentation model that uses atrous spatial pyramid pooling.
- **OCRNet [17]**: A model that incorporates object-contextual representations for semantic segmentation.
- **Attention UNet [18]**: A variant of UNet that incorporates attention mechanisms to enhance feature representation.
- **Hybrid-Segmentor [37]**: A hybrid model combining Convolutional Neural Networks (CNNs) for local feature extraction and Transformers for global feature extraction, designed for fine-grained crack segmentation in civil infrastructure.
- **CT-crackseg [19]**: A model that combines 3D CT scan data and a Transformer architecture for crack segmentation.
- **DeepCrack [35]**: A deep learning-based model for crack segmentation that uses hierarchical feature learning.
- **SECrackSeg**: Our proposed model, which integrates the Segment Anything Model 2 (SAM2) with a lightweight adapter and edge-aware attention mechanism.

Table 1 illustrates the performance comparison of various cutting-edge methods on the CFD dataset, a relatively small dataset. Given its limited sample quantity, the CFD dataset poses significant challenges for models aiming to achieve high performance. With insufficient data for training and validation, many models struggle to generalize well, often leading to overfitting or poor performance on unseen data. However, our proposed SECrackSeg model shows remarkable adaptability in this low-data scenario.

These methods are evaluated via four metrics: Precision (P), Recall (R), F1-Score (F1), and Mean Intersection over Union (mIoU). Results show that SECrackSeg outperforms other methods in all four metrics. Specifically, SECrackSeg achieves a Precision of 0.895, Recall of 0.938, F1-Score of 0.915, and mIoU of 0.854. This outstanding performance can be attributed to its innovative architecture, which includes components like the SAM2 S-Adapter. The S-Adapter allows the model to leverage the pre-trained knowledge of the large-scale SAM2 model while adapting to the specific characteristics of the small-sample CFD dataset. It effectively extracts multi-scale features related to cracks, enhancing the model’s ability to accurately segment cracks even with limited data.

The second-best method is DeepCrack, with a Precision of 0.809, Recall of 0.871, F1-Score of 0.837, and mIoU of 0.751. Other methods, including UNet, UNet++, DeeplabV3+, OCRNet, AttentionUnet, and CT-crackseg, display relatively lower performance across these metrics. Notably, CT-crackseg has a high Recall of 0.897 but a relatively low Precision of 0.717, indicating it effectively identifies positive samples while having a higher false positive rate.

Overall, the results highlight SECrackSeg’s superior performance in crack segmentation tasks on the CFD dataset, even with its limited sample size. This not only validates the effectiveness of the proposed model but also demonstrates its potential for applications where data is scarce, such as in-field inspections of infrastructure where collecting a large number of samples may be difficult or costly.

**Figure 5:**
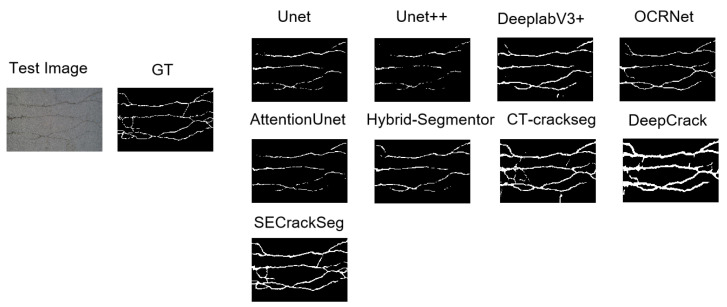
Visual comparison of crack segmentation results on a sample from the CFD dataset.

To visually demonstrate the performance of our method, we selected a sample image from the CFD dataset, as shown in Figure 5. Our method shows significant improvements in delineating the crack boundaries more accurately and capturing finer details compared to other methods. This visual comparison highlights the effectiveness of our proposed method in crack segmentation tasks.

To further assess the performance of the proposed SECrackSeg model, experiments were conducted on the Crack500 dataset. As a relatively large-scale dataset, Crack500 allows for a comprehensive evaluation of various methods. Results are presented in Table 2. As shown, SECrackSeg achieves the highest values in all four metrics, highlighting its superior performance in crack segmentation tasks. Specifically, it reaches a Precision of 0.895, Recall of 0.890, F1-Score of 0.892, and mIoU of 0.838—values significantly higher than other methods. Notably, though SECrackSeg’s Recall is slightly lower than CT-crackseg (0.908) and DeepCrack (0.895), suggesting room for improvement in specific aspects, it still dominates in overall performance.

As shown in Table 3, on the DeepCrack dataset, SECrackSeg achieves the highest values in all four metrics, demonstrating its superior performance in crack segmentation. Specifically, it reaches a Precision of 0.895, Recall of 0.900, F1-Score of 0.897, and mIoU of 0.835—significantly higher than other methods. Notably, though SECrackSeg’s recall is slightly lower than that of CT-crackseg (0.902) and DeepCrack (0.895), it still excels in overall performance. The DeepCrack dataset, with its variety of crack types and scenarios, enables a comprehensive performance evaluation of the model. Results show SECrackSeg has a strong ability to handle different crack types, making it a reliable choice for crack segmentation tasks.

#### 3.3.1. Ablation Study on the Middle Dimension of S-Adapter

To evaluate the impact of the internal bottleneck dimension in the SAM2 S-Adapter, we conducted an ablation study by varying the middle dimension $\dim_{\mathrm{mid}}$ across several settings (16, 64), while keeping the input and output dimensions fixed according to the SAM2 Hiera encoder.

In the S-Adapter module, the dimensional configurations of the linear layers play a crucial role. The linear down-projection layer, denoted as $Linear_{down}\left(dim_{in},dim_{mid}\right)$, compresses the input features from the $dim_{in}$ dimension to the $dim_{mid}$ dimension. This reduces the feature complexity and computational cost, allowing the model to focus on the key information. The linear up-projection layer, $Linear_{up}\left(dim_{mid},dim_{out}\right)$, then restores the dimension of the compressed and transformed features back to the $dim_{out}$ dimension, which is typically set to be equal to $dim_{in}$ to ensure compatibility with subsequent network layers.

Here:

- **$\dim_{\mathrm{in}}$** and **$\dim_{\mathrm{out}}$** are fixed and determined by the corresponding block of the Hiera encoder. These values are set to the number of channels at each feature scale, depending on which encoder stage the adapter is inserted.
- **$\dim_{\mathrm{mid}}$** is the only tunable hyperparameter. It represents the projection dimension within the adapter and controls its transformation capacity. A larger $\dim_{\mathrm{mid}}$ means that the linear down-projection and up-projection layers have more parameters, which can potentially introduce more expressive power. However, this may also increase the risk of overfitting and the training cost, as more complex transformations might lead the model to memorize the training data rather than learning general patterns.

**Figure 6:**
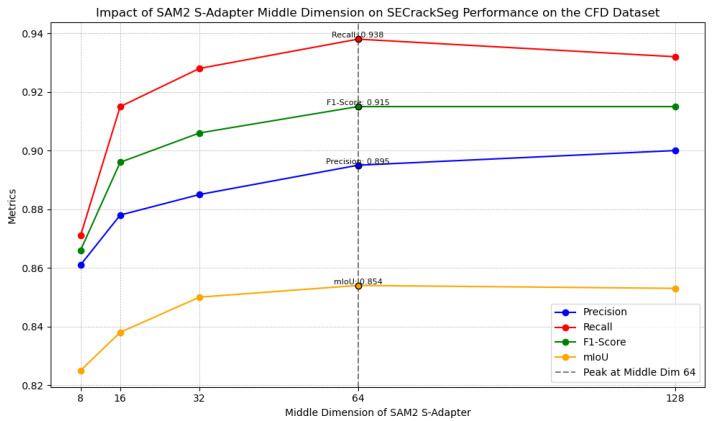
Impact of SAM2 S-Adapter middle dimension on SECrackSeg performance on the CFD dataset.

The experimental results are summarized in Figure 6, showing how different values of $\dim_{\mathrm{mid}}$ affect the performance of SECrackSeg on the CFD dataset:

- **Precision**: Increases with dimension increase. It reaches 0.895 at 64 dimensions. The increase shows that the linear layers in the S-Adapter can capture more complex feature relationships.
- **Recall**: Peaks at 0.938 with 64 dimensions. When $dim_{mid}$ changes from 16 to 64, Recall improves, but a further increase might lead to overfitting issues as seen in the potential drop at higher values.
- **F1-Score**: Reaches a maximum of 0.915 at 64. This indicates a good balance in feature transformation at this dimension.
- **mIoU**: Also reaches its highest value of 0.854 at 64 dimensions. The performance at this dimension shows the model’s ability to segment cracks accurately across all classes.

These results demonstrate that while increasing the internal bottleneck dimension can enhance the learning of complex feature transformations, it may also reduce generalization, especially on small datasets. The 64-dimensional setting provides a good trade-off between Precision and Recall, enabling accurate and robust crack segmentation.

In conclusion, $\dim_{\mathrm{mid}}=64$ is selected as the optimal configuration in the final model. This setting ensures that the adapter modules’ linear layers can effectively adapt pretrained SAM2 features while maintaining high generalization in data-scarce conditions.

#### 3.3.2. Ablation Study on the MSDC Module

**Figure 7:**
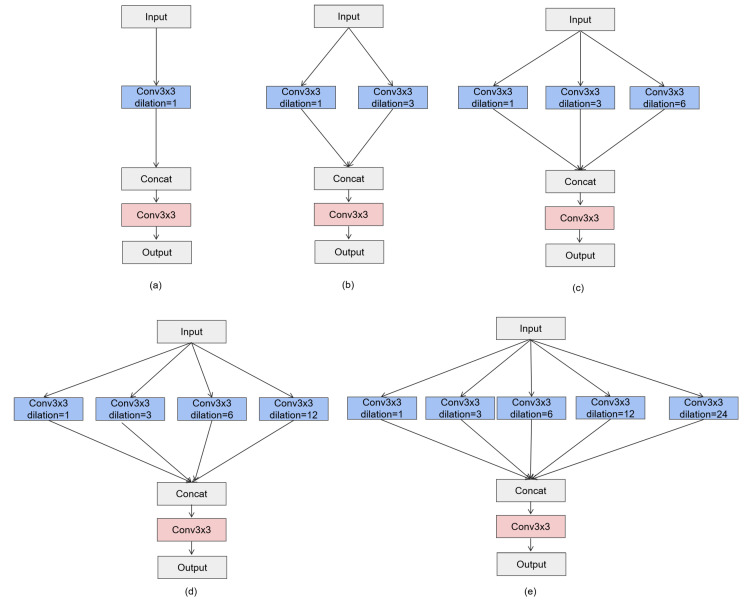
Different numbers of dilation convolution layers MSDC on the CFD dataset. They are listed as: (**a**) MSDC module with one convolutional layer (dilation rates: 1); (**b**) MSDC module with two convolutional layers (dilation rates: 1, 3); (**c**) MSDC module with three convolutional layers (dilation rates: 1, 3, 6); (**d**) MSDC module with four convolutional layers (dilation rates: 1, 3, 6, 12); (**e**) MSDC module with five convolutional layers (dilation rates: 1, 3, 6, 12, 24).

As shown in Figure 7, ablation experiments were conducted on the CFD dataset with different configurations of the MSDC module. The results are summarized in Table 4.

The MSDC module significantly enhances model performance, particularly in multi-scale feature extraction and segmentation accuracy. Looking at the results in detail, for the Precision metric, it increases steadily from 0.832 in MSDC(a) to 0.895 in MSDC(e), indicating that the model becomes more accurate in identifying truly positive samples as the number of convolutional layers and dilation rates increase. Recall also shows a rising trend, from 0.775 in MSDC(a) to 0.938 in MSDC(e), meaning the model is better at recognizing all actual positive samples. The F1-Score and mIoU follow similar patterns, with continuous improvements.

The substantial improvements from MSDC(a) to MSDC(d) clearly demonstrate the module’s effectiveness in capturing features at different scales, which is crucial for accurate segmentation. The slight improvement from MSDC(d) to MSDC(e) indicates that the module’s design is highly optimized, and additional layers may not significantly boost performance.

In practical applications such as structural health monitoring, cracks of different sizes pose varying threats to the integrity of structures. The MSDC module’s ability to accurately segment cracks of different scales is of great significance. It enables the detection of both small and large cracks, helping engineers to identify potential safety hazards in a timely manner and make appropriate maintenance decisions. Overall, the results show that the MSDC module effectively improves the model’s ability to capture multi-scale features, thereby enhancing segmentation accuracy on the CFD dataset.

#### 3.3.3. Ablation Study on the MI-Upsampling Module

To comprehensively evaluate the impact of the MI-Upsampling module in the SECrackSeg model, we conducted ablation experiments comparing it with traditional upsampling methods, including bilinear interpolation and transposed convolution. The results are shown in Table 5.

As shown in Table 5, the MI-Upsampling module achieves the best performance across all evaluation metrics—Precision, Recall, F1-Score, and mIoU. While the absolute improvements over bilinear interpolation and transposed convolution may appear relatively small, these gains are consistent and indicate meaningful enhancements in edge prediction and spatial detail preservation.

Notably, the Recall improves from 0.912 (bilinear) and 0.925 (transposed convolution) to 0.938 with MI-Upsampling, reflecting better crack completeness. Meanwhile, the F1-Score rises to 0.915 and mIoU to 0.854, confirming improved balance between Precision and Recall.

Beyond numerical performance, MI-Upsampling is explicitly designed to address two critical limitations of traditional upsampling: (1) information loss and (2) the generation of pseudo-edges. It integrates slice-based, bilinear, and deconvolution strategies in a hybrid fusion scheme to enhance structural details while minimizing artifacts.

**Figure 8:**
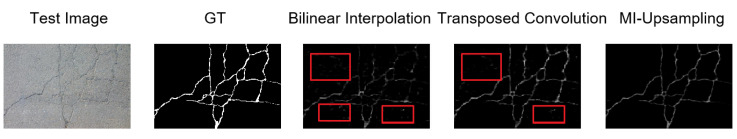
Visual comparison of different upsampling methods on a CFD sample. Red boxes highlight pseudo-edge artifacts generated by bilinear interpolation and transposed convolution. MI-Upsampling significantly reduces these artifacts while preserving edge continuity.

To better illustrate these improvements, we provide a visual comparison in Figure 8. As shown, MI-Upsampling produces cleaner crack boundaries and suppresses pseudo-edges (highlighted in red), which are frequently present in outputs from the other two methods.

In summary, although MI-Upsampling yields moderate metric improvements, its architectural advantages—enhanced edge fidelity, detail preservation, and suppression of pseudo-edges—make it a vital component of the SECrackSeg pipeline. These enhancements contribute meaningfully to the model’s robustness and segmentation quality, particularly in challenging real-world crack scenarios.

#### 3.3.4. Ablation Study on the Edge-Aware Attention Module

As shown in Table 5, ablation experiments were conducted on the CFD dataset with different configurations of the Edge-Aware Attention Module:

- **Method 1**: Output from the last decoder without any Edge-Aware Attention Modules.
- **Method 2**: One Edge-Aware Attention Module connected to the last decoder.
- **Method 3**: Two Edge-Aware Attention Modules connected to the last two decoders.
- **Method 4**: Three Edge-Aware Attention Modules connected to the last three decoders.

The results in Table 6 demonstrate the impact of using different numbers of Edge-Aware Attention Modules on the model’s performance. Method 1, with a single Edge-Aware Attention Module, shows a noticeable improvement in all metrics compared to not using the module. Method 2, which uses two modules, further enhances the performance, indicating that capturing edge details at multiple stages is beneficial. Method 3, with three modules, achieves the highest scores across all metrics, highlighting the effectiveness of the Edge-Aware Attention Module in improving segmentation accuracy, especially for fine-grained edge details

#### 3.3.5. Ablation Study on Loss Function

As shown in Table 7, ablation experiments were conducted on the CFD dataset with different configurations of the loss function.

The model’s loss function combines weighted binary cross-entropy ($L_{BCEw}$) and weighted IoU loss ($L_{IoUw}$) with multi-granularity supervision. Comparing the standard binary cross-entropy loss ($L_{BCE}$), standard IoU loss ($L_{IoU}$), the proposed weighted combination ($L=L_{IoUw}+L_{BCEw}$), and the total loss with multi-granularity supervision ($L_{total}=\sum_{i=1}^{3}L\left(G_{i},S_{i}\right)$), the results show that the standard losses have lower performance. The weighted combination improves performance by emphasizing difficult pixels, and the total loss with multi-granularity supervision achieves the best results, with the highest Precision, Recall, F1-Score, and mIoU values. This demonstrates the effectiveness of the designed loss function in enhancing crack segmentation accuracy.

#### 3.3.6. Ablation Study from Baseline to SECrackSeg

As shown in Table 8, the baseline U-Net model achieves a Precision of 0.855, Recall of 0.716, F1-Score of 0.779, and mIoU of 0.680 on the CFD dataset. The integration of the **SAM2 S-Adapter** significantly enhances performance across all metrics: Precision improves to 0.872 (**+1.7%**), Recall to 0.803 (**+8.7%**), F1-Score to 0.836 (**+5.7%**), and mIoU to 0.820 (**+14.0%**).This significant enhancement in the small-sample scenario indicates that the model can better extract features from limited data.

Subsequently, the **MSDC module** demonstrates substantial improvements in multi-scale feature fusion: Precision increases to 0.887 (**+1.5%**), Recall jumps to 0.860 (**+5.7%**), F1-Score reaches 0.873 (**+3.7%**), and mIoU rises to 0.838 (**+1.8%**). These gains highlight its critical role in capturing diverse crack scales through dilated convolutions.

The **MI-Upsampling** module then provides modest refinement: Precision slightly improves to 0.890 (**+0.3%**), Recall enhances to 0.875 (**+1.5%**), F1-Score achieves 0.882 (**+0.9%**), and mIoU stabilizes at 0.842 (**+0.4%**), validating its role in preserving structural details.

Finally, the **Edge-Aware Attention mechanism** elevates performance to peak levels: Precision peaks at 0.895 (**+0.5%**), Recall surges to 0.938 (**+6.3%**), F1-Score culminates at 0.915 (**+3.3%**), and mIoU reaches 0.854 (**+1.2%**), demonstrating exceptional edge segmentation capability.

## 4. Conclusions

The results demonstrate the effectiveness of SECrackSeg in addressing key challenges in crack segmentation, including generalization, fine-grained feature extraction, and edge detail capture. These findings are discussed in the context of previous studies and the working hypotheses.

### 4.1. Interpretation of Results

The SAM2 S-Adapter significantly enhances the model’s generalization ability, as evidenced by improved performance on all datasets. This supports our hypothesis that a frozen backbone and lightweight adapter design improves robustness in complex and noisy environments. The MSDC module effectively captures multi-scale features, enabling more accurate segmentation of cracks of varying scales. MI-Upsampling, by integrating slice-based upsampling, bilinear interpolation, and deconvolution, preserves edge and detail information effectively. The Edge-Aware Attention mechanism further boosts the model’s ability to focus on boundary features, leading to improved mIoU and F1-Score performance.

To further evaluate computational efficiency and training cost, we benchmarked several crack segmentation models on the CFD dataset. All models were trained under the same conditions: 500 epochs, identical input size (480 × 320), and batch size, using a Windows 10 system with an RTX 2070 Super GPU. We report training time, inference time, parameter count, and floating-point operations (FLOPs) in Table 9.

As shown in Table 9, SECrackSeg has the highest inference time and training time among all models due to its multi-stage design and the integration of a large pretrained SAM2 backbone. However, this additional cost leads to significantly better segmentation performance. Lightweight models such as UNet and DeepLabv3+ converge quickly within 1–1.5 h, while other complex models like Hybrid_Segmenter and CT-CrackSeg fall between in terms of training cost and complexity. Although OCRNet has fewer parameters, its multi-branch structure and high FLOPs lead to moderately longer training time.

These results demonstrate that while SECrackSeg is not the fastest, it achieves a favorable trade-off between accuracy and computational cost, especially in offline analysis scenarios where precision is more critical than speed. Furthermore, its strong performance on the CFD dataset—despite data scarcity—confirms the effectiveness of the SAM2-based transfer learning strategy and the lightweight fine-tuning capability of the S-Adapter module.

### 4.2. Implications

SECrackSeg’s superior performance has significant implications for structural health monitoring and infrastructure maintenance, enabling early detection of structural issues and reducing safety risks. The integration of SAM2, MSDC, and Edge-Aware Attention provides a new framework for crack segmentation, which can be adapted to other image segmentation tasks.

### 4.3. Future Research Directions

Exploring data augmentation techniques and synthetic data could improve generalization and robustness. Extending SECrackSeg to handle related tasks such as crack width estimation and crack type classification would broaden its application scope. Optimizing the model for real-time applications through lightweight design and hardware acceleration would enhance its practicality. Investigating the model’s performance in diverse scenarios would further enhance its generalization capabilities.

## Figures and Tables

**Table 1:** Comparison of state-of-the-art methods on the CFD dataset.

Methods	Resolution	Num (Train/Test)	P	R	F1	mIoU
UNet	480 × 320	118 (83/35)	0.855	0.716	0.779	0.680
UNet++	480 × 320	118 (83/35)	0.849	0.660	0.718	0.635
DeeplabV3+	480 × 320	118 (83/35)	0.810	0.820	0.815	0.727
OCRNet	480 × 320	118 (83/35)	0.816	0.858	0.836	0.750
AttentionUnet	480 × 320	118 (83/35)	0.843	0.732	0.776	0.688
Hybrid-Segmentor	480 × 320	118 (83/35)	0.723	0.738	0.730	0.657
CT-crackseg	480 × 320	118 (83/35)	0.717	0.897	0.789	0.698
DeepCrack	480 × 320	118 (83/35)	0.809	0.871	0.837	0.751
SECrackSeg	480 × 320	118 (83/35)	0.895	0.938	0.915	0.854

**Table 2:** Comparison of state-of-the-art methods on the Crack500 dataset.

Methods	Resolution	Num (Train/Test)	P	R	F1	mIoU
UNet	640 × 360	3368 (2358/1010)	0.839	0.743	0.787	0.685
UNet++	640 × 360	3368 (2358/1010)	0.869	0.720	0.787	0.677
DeeplabV3+	640 × 360	3368 (2358/1010)	0.851	0.857	0.854	0.767
OCRNet	640 × 360	3368 (2358/1010)	0.857	0.865	0.861	0.776
AttentionUnet	640 × 360	3368 (2358/1010)	0.890	0.831	0.859	0.773
Hybrid-Segmentor	640 × 360	3368 (2358/1010)	0.832	0.848	0.841	0.755
CT-crackseg	640 × 360	3368 (2358/1010)	0.785	0.908	0.842	0.758
DeepCrack	640 × 360	3368 (2358/1010)	0.854	0.895	0.874	0.791
SECrackSeg	640 × 360	3368 (2358/1010)	0.895	0.890	0.892	0.838

**Table 3:** Comparison of state-of-the-art methods on the DeepCrack dataset.

Methods	Resolution	Num (Train/Test)	P	R	F1	mIoU
UNet	544 × 384	537 (376/161)	0.812	0.798	0.805	0.725
UNet++	544 × 384	537 (376/161)	0.847	0.785	0.815	0.733
DeeplabV3+	544 × 384	537 (376/161)	0.868	0.889	0.879	0.802
OCRNet	544 × 384	537 (376/161)	0.882	0.901	0.892	0.823
AttentionUnet	544 × 384	537 (376/161)	0.903	0.862	0.882	0.804
Hybrid-Segmentor	544 × 384	537 (376/161)	0.845	0.870	0.857	0.765
CT-crackseg	544 × 384	537 (376/161)	0.830	0.902	0.865	0.779
DeepCrack	544 × 384	537 (376/161)	0.890	0.895	0.893	0.830
SECrackSeg	544 × 384	537 (376/161)	0.895	0.900	0.898	0.835

**Table 4:** Ablation study on the MSDC module on the CFD dataset.

Methods	Resolution	Num (Train/Test)	P	R	F1	mIoU
MSDC(a)	480 × 320	118 (83/35)	0.832	0.775	0.803	0.741
MSDC(b)	480 × 320	118 (83/35)	0.862	0.810	0.835	0.763
MSDC(c)	480 × 320	118 (83/35)	0.875	0.865	0.870	0.814
MSDC(d)	480 × 320	118 (83/35)	0.890	0.915	0.902	0.848
MSDC(e)	480 × 320	118 (83/35)	0.895	0.938	0.915	0.854

**Table 5:** Ablation study on the MI-Upsampling module on the CFD dataset.

Methods	Resolution	Num (Train/Test)	P	R	F1	mIoU
Bilinear Interpolation	480 × 320	118 (83/35)	0.882	0.912	0.897	0.847
Transposed Convolution	480 × 320	118 (83/35)	0.891	0.925	0.904	0.850
MI-Upsampling	480 × 320	118 (83/35)	0.895	0.938	0.915	0.854

**Table 6:** Ablation study on the Edge-Aware Attention Module on the CFD dataset.

Methods	Resolution	Num (Train/Test)	P	R	F1	mIoU
Method 1	480 × 320	118 (83/35)	0.853	0.830	0.841	0.830
Method 2	480 × 320	118 (83/35)	0.877	0.904	0.890	0.839
Method 3	480 × 320	118 (83/35)	0.891	0.926	0.908	0.851
Method 4	480 × 320	118 (83/35)	0.895	0.938	0.915	0.854

**Table 7:** Ablation study on the loss function on the CFD dataset.

Methods	Resolution	Num (Train/Test)	P	R	F1	mIoU
$L_{\mathrm{BCE}}$	480 × 320	118 (83/35)	0.842	0.820	0.831	0.829
$L_{\mathrm{IoU}}$	480 × 320	118 (83/35)	0.856	0.858	0.857	0.832
$L=L_{\mathrm{IoU}w}+L_{\mathrm{BCE}w}$	480 × 320	118 (83/35)	0.878	0.895	0.886	0.847
$L_{\mathrm{total}}=\sum_{i=1}^{3}L\left(G_{i},S_{i}\right)$	480 × 320	118 (83/35)	0.895	0.938	0.915	0.854

**Table 8:** Ablation study results from baseline to SECrackSeg on the CFD dataset.

Method	Resolution	Num (Train/Test)	P	R	F1	mIoU
UNet (Baseline)	480 × 320	118 (83/35)	0.855	0.716	0.779	0.680
SAM2 S-Adapter (V1)	480 × 320	118 (83/35)	0.872	0.803	0.836	0.820
MSDC Module (V2)	480 × 320	118 (83/35)	0.887	0.860	0.873	0.838
MI-Upsampling (V3)	480 × 320	118 (83/35)	0.890	0.875	0.882	0.842
Edge-Aware Attention (V4)	480 × 320	118 (83/35)	0.895	0.938	0.915	0.854

**Table 9:** Comparison of inference time, training time, and model complexity for different crack segmentation models on the CFD dataset (trained for 500 epochs, input size: 480 × 320).

Method	Resolution	Inference Time (s)	Training Time (h)	Params Size (M)	FLOPs (G)
UNet	480 × 320	0.0296	1.2	13.40	72.83
UNet++	480 × 320	0.0370	1.4	8.37	70.12
DeepLabv3+	480 × 320	0.0271	1.0	6.27	23.02
OCRNet	480 × 320	0.0821	1.6	0.67	94.91
AttentionUNet	480 × 320	0.0248	1.5	31.40	261.78
Hybrid_Segmenter	480 × 320	0.1716	6.8	226.81	628.50
CT-CrackSeg	480 × 320	0.2250	3.2	22.88	93.94
DeepCrack	480 × 320	0.0286	1.3	14.72	94.33
SECrackSeg	480 × 320	1.5762	12.9	223.49	329.56

## Data Availability

The original data presented in the study are openly available in the following publicly accessible repositories: CFD dataset: https://github.com/cuilimeng/CrackForest-dataset (accessed on 1 January 2025); Crack500 dataset: https://github.com/fyangneil/pavement-crack-detection (accessed on 1 January 2025); DeepCrack dataset: https://github.com/yhlleo/DeepCrack (accessed on 1 January 2025).
